# Vibrational Quantum-State-Controlled Reactivity in the O_2_^+^ + C_3_H_4_ Reaction

**DOI:** 10.1021/acs.jpclett.6c00425

**Published:** 2026-04-06

**Authors:** C. Zagorec-Marks, G. S. Kocheril, T. Kieft, O. A. Krohn, C. Martí, T. P. Softley, J. Zádor, H. J. Lewandowski

**Affiliations:** † Department of Physics, 172322 University of Colorado, Boulder, Colorado 80309, United States; ‡ JILA, National Institute of Standards and Technology and the University of Colorado, Boulder, Colorado 80309, United States; ¶ Combustion Research Facility, 1877 Sandia National Laboratories, Livermore, California 94550, United States; § School of Chemistry, 111651 University of Birmingham, Edgbaston B15 2TT, U.K.

## Abstract

Quantum-state-controlled reactivity is a long-standing goal in the field of physical chemistry. In this work, we explore the vibrational-state-dependent behavior of the ion–molecule reaction between O_2_^+^ in distinct vibrational states and two isomers of C_3_H_4_, allene (H_2_C_3_H_2_) and propyne (H_3_C_3_H). While most products are formed regardless of the vibrational state of O_2_^+^, the branching ratios are influenced by vibrational excitation, and a new product, C_2_O^+^, appears exclusively in the excited-state reactions. This selective formation of C_2_O^+^ demonstrates that vibrational excitation can effectively activate a reaction pathway, providing direct evidence of quantum-state control in the reactivity. These results represent an important step toward the goal of quantum-state-controlled chemistry in molecular systems.

## Graphical abstract

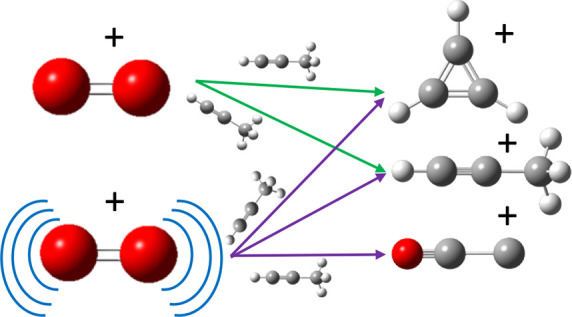

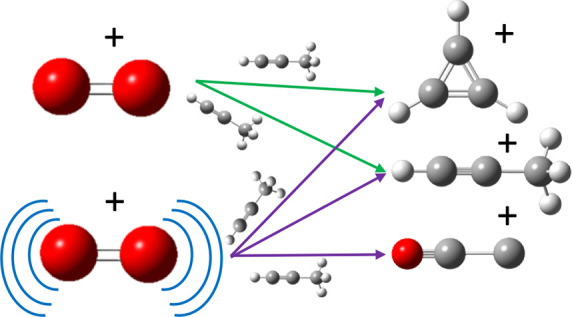

Understanding the role of individual quantum states on reactivity is a critical component to developing control of chemical reaction processes, which is an often-stated goal for the physical chemistry community.^1^ Original endeavors to achieve this goal investigated the effects of individual quantum states in atom–diatomic reactions and resulted in the development of Polanyi’s rules.^2–5^ With these rules, Polanyi suggested that not all forms of energy (vibrational, translational, etc.) are equivalent in determining the progression of a reaction. Specifically, as observed in these early studies, translational energy facilitated an atom–diatom reaction’s progression through an early barrier (i.e., a barrier located along the approach coordinate of reaction), whereas vibrational energy facilitated progression through a late barrier (i.e., a barrier located along the separation coordinate). Additionally, it was seen that the energy distribution of the reaction products depended on the position of the barrier, with an early barrier promoting greater partitioning of energy into the products’ vibrational modes and a late barrier promoting greater partitioning into the products’ translational energy.

Additional experimental support for Polanyi’s rules was provided with the advent of lasers. This development renewed interest in state-dependent chemistry, as quantum states of reactants could be directly populated in atom–diatomic reaction systems.^6^ However, as studies expanded to polyatomic systems, it was found that Polanyi’s rules were often not sufficient, particularly for ion–molecule reactions in which submerged barriers are commonly present.^7–12^ More recent theoretical efforts have extended Polanyi’s rules to be more applicable to polyatomic reactions through the use of quasi-classical trajectory simulations, quantum dynamics calculations, and sudden vector projection models.^13–19^

Although there has been a significant amount of theoretical progress in exploring state-dependent reactivity,^1–5,13–19^ there has been less experimental progress.^18,20–29^ This is due to the experimental difficulty in preparing reactants in a pure quantum state and ensuring that these reactants do not decay to their ground state or, in the case of polyatomic molecules, disperse the excitation energy via intramolecular vibrational redistribution (IVR) prior to the reaction.

Ion–molecule reactions are especially well suited for studies of quantum-state-controlled chemistry, since they are typically exothermic and barrierless. Such reactions proceed rapidly (*k* ∼ 10^–9^, cm^3^ s^–1^)^30^ and often occur too quickly for significant redistribution of energy among different internal states. In particular, homonuclear diatomic ions are ideal for studying vibrational-state-dependent reactivity because their lack of an electric dipole moment can result in long-lived, excited vibrational states, and they are immune to IVR by having only one fundamental vibrational mode.^31,32^ O_2_^+^ is an apt candidate for such studies, as the vibrational lifetime of the X^2^Π_g_ state has been calculated to be as long as 5.7 × 10^5^ s for the *v* = 30 vibrational mode, with even longer lifetimes predicted for lower-lying states, as their decay proceeds solely through quadrupole emission.^33^

Previously, we investigated reactions of O_2_^+^ in the X^2^Π_g_ vibrationally excited states (*v* = 2, 3) with the C_3_H_4_ isomers, allene (H_2_C_3_H_2_) and propyne (H_3_C_3_H), to elucidate the influence of isomeric structure on reactivity. In that study, a product with a mass-to-charge ratio of 40 *m*/*z* was found to be nonreactive with C_3_H_4_, which provided evidence that it may be a product other than C_3_H_4_^+^, which is known to react with C_3_H_4_.^34^ C_2_O^+^ was previously proposed as the identity of this nonreactive 40 *m*/*z* based on the lack of reactivity and the reaction’s atomic composition. The possible production of C_2_O^+^ in that study, in addition to the knowledge that O_2_^+^ had been formed in a mixture of vibrationally excited states (*v* = 2, 3), encouraged our further exploration into how vibrational excitation may influence this reaction. Here, we explored the effects of vibrational excitation of O_2_^+^ on the ion–molecule reactions, shown below, between O_2_^+^ and the C_3_H_4_ isomers.

1 $${O_{2}}^{+}\left(v=0\right)+C_{3}H_{4}\to ?$$

2 $${O_{2}}^{+}\left(v=2,3\right)+C_{3}H_{4}\to ?$$

We found that the vibrational state of O_2_^+^ governs the formation of the nonreacting 40 *m*/*z* product, which is reasoned to be C_2_O^+^. This product is observed only upon vibrational excitation of O_2_^+^ despite calculations predicting the feasibility of its production in the ground state. Thus, this work demonstrates that the reaction of allene and propyne with O_2_^+^ in different vibrational states impacts the dynamics by activating or deactivating reaction pathways and ultimately the observed reaction products.

The remainder of this Letter is organized as follows. First, a brief description of the experimental apparatus is provided, so that the results can be more readily interpreted. This is followed by a short discussion of previous experimental results for the reactions of O_2_^+^,^34^ in the *v* = (2, 3) excited vibrational states, with both C_3_H_4_ isomers, allene (H_2_C_3_H_2_) and propyne (H_3_C_3_H). Next, new data for the reactions of O_2_^+^, in the ground vibrational state, with both C_3_H_4_ isomers are presented and discussed. Finally, these results are compared, thereby demonstrating how O_2_^+^’s vibrational state affects the progression of these reactions.

The experimental apparatus is summarized here (with full details in the Methods section) to provide context for the results. The experiment uses atomic-physics techniques to achieve single-collision conditions and ion translational temperatures below 10 K, where temperature here refers to a spread in energy and is not representative of a true temperature of a Maxwell–Boltzmann distribution.

To achieve these conditions, laser-cooled Ca^+^ ions are confined in a linear, quadrupole ion trap within an ultrahigh-vacuum chamber, forming a pseudocrystalline structure known as a Coulomb crystal, which sympathetically cools cotrapped molecular ions through Coulomb interactions. The O_2_^+^ ions are created by two separate (2 + 1) resonance enhanced multiphoton ionization (REMPI) schemes, which create ions predominantly in the ground state (∼91% in ground state) or a mixture of excited vibrational states (*v* = 2 and 3, ∼68% and ∼28% of ions, respectively).^35–38^ Because the ions are separated by ∼10 μm, this sympathetic cooling acts on only translational motion and does not impact vibrational-state populations. Combined with the ultrahigh-vacuum environment, which leads to a low rate (<1 Hz) of background gas collisions, this ensures that O_2_^+^ remains in its initial vibrational state until a reactive collision occurs. The vacuum conditions further preclude termolecular processes; therefore, internal energy in reaction complexes can dissipate only through bimolecular dissociation or radiative decay.

Reactions are initiated by introducing neutral C_3_H_4_ into the vacuum system at pressures that result in collision rates on the order of ∼1 Hz. After C_3_H_4_ is admitted for a set amount of time, the trap contents (which include all charged reactants and products) are extracted into a time-of-flight mass spectrometer. Reaction curves are obtained by repeating this procedure with a range of reaction times.

We first consider previous results where vibrationally excited O_2_^+^ (*v* = 2, 3) reacted with two C_3_H_4_ isomers.^34^ The reaction curves from that work are shown in panels (b) and (d) of Figure 1. In these reactions, we observed that the primary product formed was *c*-C_3_H_3_^+^ (teal points), which accounted for ∼73% (∼130 ions) of all products in the reaction of O_2_^+^ with allene (d) and ∼62% (∼110 ions) of all products in the reaction of O_2_^+^ with propyne (b).

**Figure 1:**
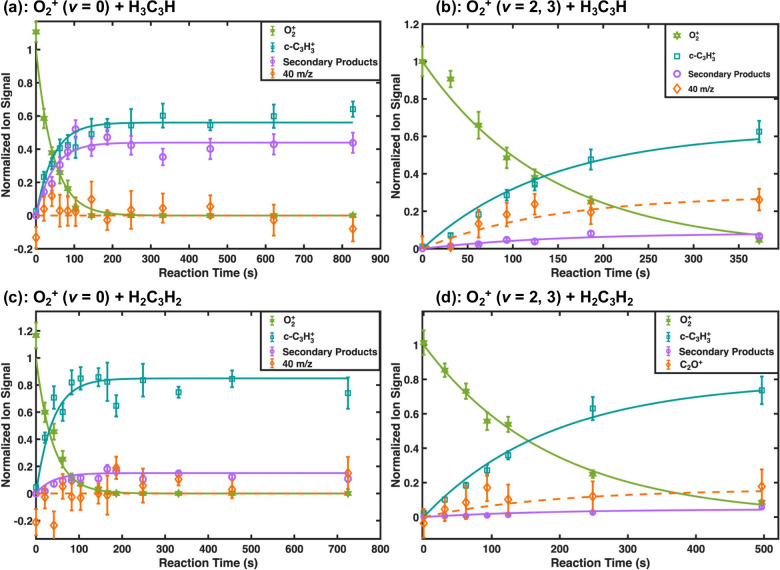
Reaction curves for (a) O_2_^+^ (*v* = 0) + H_3_C_3_H (propyne), (b) O_2_^+^ (*v* = 2, 3) + H_3_C_3_H, (c) O_2_^+^ (*v* = 0) + H_2_C_3_H_2_ (allene), and (d) the O_2_^+^ (*v* = 2, 3) + H_2_C_3_H_2_ reaction series. As the O_2_^+^ signal decreases (green), immediate growth is observed in the 39 *m*/*z* (teal) and 40 *m*/*z* (orange) mass channels. The product signal in the 40 *m*/*z* channel was determined from conservation of charge because of the mass overlap of this product with the trapped Ca^+^. Delayed growth is observed in the 77 and 79 *m*/*z* secondary product channels (combined into one curve displayed in purple). The appearance times, mass-to-charge ratios, and stability of these channels identifies these products as c-C_3_H_3_^+^ (39 *m*/*z*), C_2_O^+^/C_3_H_4_^+^ (40 *m*/*z*), C_6_H_5_^+^ (77 *m*/*z*) and C_6_H_7_^+^ (79 *m*/*z*). c-C_3_H_3_^+^, C_2_O^+^, and C_3_H_4_^+^ are primary products and the C_6_H_*y*_^+^ (*y* = 5, 7) ions are secondary products originating from the reaction C_3_H_4_^+^ + C_3_H_4_. Ion signals have been normalized to the fitted initial number of O_2_^+^ ions. Error bars correspond to 1σ standard error. C_3_H_4_ was admitted at a partial pressure of ∼1.2 × 10^–9^ and ∼0.3 × 10^–10^ Torr for the *v* = 0 and *v* = (2, 3) reaction, respectively. Panels (b) and (d) reproduced from ref 34 with permission from the Royal Society of Chemistry.

Because *c*-C_3_H_3_^+^ has been observed to be far less reactive than its linear counterpart with a variety of molecules and it has a *m*/*z* = 39,^39–43^ we assigned this structure to the observed 39 *m*/*z* product. In addition to *c*-C_3_H_3_^+^, we indirectly observed another product channel at 40 *m*/*z* (orange points), which accounted for ∼17% (∼30 ions) of all products in the reaction of O_2_^+^ with allene and ∼30% (∼70 ions) of all products in the reaction of O_2_^+^ with propyne. Although we would have preferred a direct measurement of this product channel in the previous study, the presence of Ca^+^ (∼700 ions) at the same mass led to a large intrinsic uncertainty in the measurement of this much smaller number of product ions. This uncertainty was mostly due to the natural shot-to-shot variations in the loading of Ca ions into the trap (±50 ions), which are comparable in magnitude to the product signal. To reduce the uncertainty arising from the substantial Ca^+^ background, we used an indirect method to determine the product ions at 40 *m*/*z*.

For this indirect method to be valid, two conditions had to be met. First, the total number of ions in the trap needed to remain constant over the course of the reaction. Second, the number of O_2_^+^ ions loaded into the trap also had to remain consistent, with shot-to-shot variations that were small compared to the average number of O_2_^+^ ions loaded (∼180 ions). Once these conditions were satisfied, we determined the number of product ions contributing to the 40 *m*/*z* channel using the following equation:

3 $$N_{40}\left(t\right)=N_{{O_{2}}^{+}}\left(t=0\right)-\left(N_{{O_{2}}^{+}}\left(t\right)+N_{C_{3}{H_{3}}^{+}}\left(t\right)+N_{\mathrm{Secondary}\mathrm{Products}}\left(t\right)\right)$$

where *N*_40_(*t*) is the number of ions with a *m*/*z* of 40 in the trap at time, *t*, that are not Ca^+^, *N*_O_2_^+^_(*t*) and *N*_C_3_H_3_^+^_(*t*) are the number of O_2_^+^ and C_3_H_3_^+^ ions in the trap at time *t*, and *N*_SecondaryProducts_(*t*) is the number of secondary product ions in the trap at time *t* that were produced from the reactions of C_3_H_4_^+^ with neutral C_3_H_4_.

Although the 40 *m*/*z* product could, in principle, have consisted solely of the charge-transfer product C_3_H_4_^+^, previous studies have shown that C_3_H_4_^+^ reacts with neutral C_3_H_4_ to form C_6_H_5_^+^ and C_6_H_7_^+^,^39,44^ and we observed both reactive and nonreactive —within the time scale of our experiments —components in the 40 *m*/*z* channel besides Ca^+^. The secondary products from the C_3_H_4_^+^ + C_3_H_4_ reaction (77 and 79 *m*/*z*) were measured to account for only a small portion of the total product signal (∼3%). This suggested most of the ions with 40 *m*/*z* were not C_3_H_4_^+^, and that an additional species was responsible for the nonreacting fraction. Given the atomic composition of the reactants, there were only two possible combinations of atoms that could yield *m*/*z* = 40, C_3_H_4_^+^ and C_2_O^+^. Because of this, the most reasonable assignment for the nonreactive *m*/*z* = 40 product was C_2_O^+^.

In these previously studied reactions, it appeared that the nonreacting 40 *m*/*z* product (likely C_2_O^+^) accounted for the majority of the 40 *m*/*z* product channel. Because these reactions were conducted with O_2_^+^ prepared in a mixture of vibrationally excited states, and it is known that vibrational excitations can influence product branching ratios,^45^ we revisited these reactions with O_2_^+^ prepared in the ground vibrational state to determine how the results of the reaction might be affected by vibrational excitation. The results of these new studies are presented here.

For these studies, we prepared the O_2_^+^ ions in the ground vibrational state and reacted them with both C_3_H_4_ isomers. Results from these experiments are shown in panels (a) and (c) of Figure 1, where only two primary products were observed. The majority of product ions from both reactions was the previously observed *c*-C_3_H_3_^+^ that accounted for ∼85% (∼60 ions) of all products in the reaction of O_2_^+^ with allene (c) and ∼56% (∼40 ions) of all products in the reaction of O_2_^+^ with propyne (a). Unlike the reactions with vibrationally excited O_2_^+^, the only other primary product produced was C_3_H_4_^+^, which accounted for ∼15% (∼10 ions) in the reaction of O_2_^+^ with allene and ∼44% (∼30 ions) of all products in the reaction of O_2_^+^ with propyne.

C_3_H_4_^+^ production is determined by measuring the accumulation of products from the C_3_H_4_^+^ + C_3_H_4_ reaction, which are C_6_H_5_^+^ and C_6_H_7_^+^. These secondary products can be unambiguously detected in our experiment with no overlap with Ca^+^ or any primary reaction products. If we sum the product ions (*c*-C_3_H_3_^+^, C_6_H_5_^+^, and C_6_H_7_^+^) and remaining reactant ions at any point in time along the reaction curve, we see that the sum is equal to the initial number of reactant O_2_^+^ ions, as shown in the Supporting Information Figure 1. This conservation demonstrates that, for ground state O_2_^+^, no nonreactive 40 *m*/*z* products were formed.

This behavior highlights a key distinction between the ground-state and the vibrationally excited-state reactions. In the vibrationally excited case, a significant fraction of 40 *m*/*z* products did not undergo secondary reactions with C_3_H_4_ within the time of the experiment, suggesting that two distinct products may have been present in the 40 *m*/*z* channel. In contrast, in the ground-state case, all 40 *m*/*z* products underwent secondary reactions with C_3_H_4_ indicating that this product channel consisted exclusively of a single species, C_3_H_4_^+^. This finding prompted us to confirm the presence of the nonreacting 40 *m*/*z* product through a direct measurement.

Although the production of C_2_O^+^ in the vibrationally excited case was previously suggested from charge conservation arguments,^34^ we provide additional evidence of the formation of a nonreacting product with 40 *m*/*z* here through the use of a ^44^Ca^+^ Coulomb crystal devoid of ^40^Ca^+^ ions and isotopic substitution of the reactants. To remove the large background signal from ^40^Ca^+^ in the mass spectrum, we created a Coulomb crystal of ^44^Ca^+^ devoid of ^40^Ca^+^ ions. This was achieved by first preparing a large Coulomb crystal containing approximately 3000 Ca^+^ ions with the natural isotopic abundance, and then shifting the cooling-laser frequencies to selectively address the ^44^Ca^+^ transitions.^46,47^ The ^40^Ca^+^ isotopes left the trap due to heating, either through deliberate parametric excitation or by lowering the trapping potentials to remove high-energy ions. This procedure yielded a small ^44^Ca^+^ Coulomb crystal containing approximately 70 ions. While this crystal was sufficiently large to enable product identification, it did not provide enough sympathetic cooling to ensure all product ions remained trapped throughout the full course of a reaction. Consequently, the ^44^Ca^+^ Coulomb crystal was not used to measure reaction kinetics, but instead served to identify the presence of a nonreacting 40 *m*/*z* product.

After preparing the ^44^Ca^+^ Coulomb crystal, O_2_^+^ ions were loaded into the trap in a mixture of vibrationally excited states (*v* = 2, 3). Reactions were then performed using either deuterated allene (D_2_C_3_D_2_, *m*/*z* = 44) or propyne (D_3_C_3_D, *m*/*z* = 44), which were selected to isolate growth in the 40 *m*/*z* channel and thereby support the identification of C_2_O^+^ as a reaction product.

Growth was observed in the 40 *m*/*z* channel in addition to all other expected products (c-C_3_D_3_^+^ with *m*/*z* = 42 and C_3_D_4_^+^ with *m*/*z* = 44) as shown in Figure 2. Because of the low pressures involved in this experiment, it is highly unlikely that the 40 *m*/*z* signal is C_3_DH_2_^+^ formed via multiple H–D scrambling events with H_2_O and C_3_D_3^+^_. Additional control experiments (e.g., not admitting neutral propyne/allene or not trapping O_2_^+^) confirmed that the 40 *m*/*z* growth originated from reactions between O_2_^+^ and C_3_D_4_ and not via reactions with any contaminants. Therefore, because growth in the 40 *m*/*z* channel was observed only when both O_2_^+^ in vibrationally excited states (*v* = 2, 3) and C_3_X_4_ (X = H, D) were present, this signal was assigned to the formation of C_2_O^+^.

**Figure 2:**
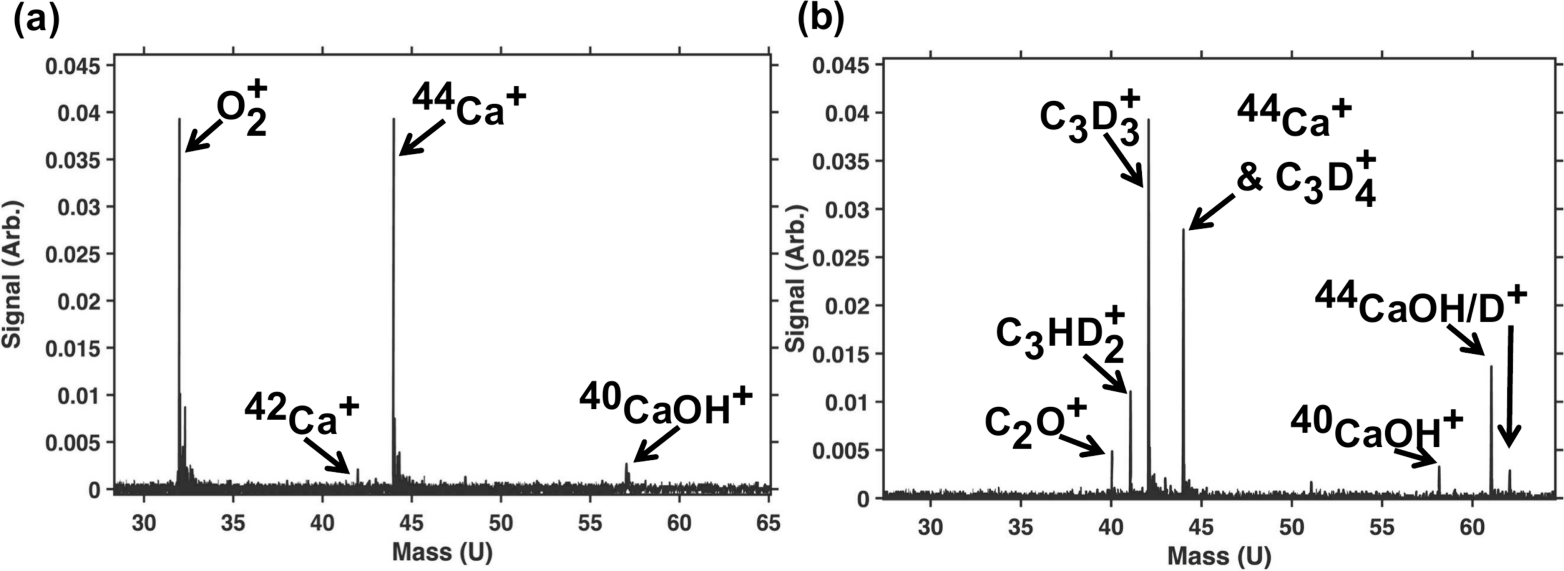
TOF-MS traces depicting the reaction of O_2_^+^ (*v* = 2,3) + C_3_D_4_ within a ^44^Ca^+^ Coulomb crystal with no ^40^Ca^+^ present. These data correspond to a reaction using propyne-*d*_4_ (D_3_C_3_D). (a) Example TOF-MS trace at *t* = 0 in which O_2_^+^ ions are observed at 32 *m*/*z* and ^44^Ca^+^ at 44 *m*/*z*. The two smaller peaks present correspond to ^42^Ca^+^ and ^40^CaOH^+^ (57 *m*/*z*) that were not removed from the crystal prior to reaction as their mass channels were not being investigated. We observe no ions in the 40 *m*/*z* location with single ion sensitivity. (b) Example of a TOF-MS trace at *t* = 300 s in which O_2_^+^ has fully reacted with C_3_D_4_ to form: C_2_O^+^ at 40 *m*/*z*, C_3_D_3_^+^ at 42 *m*/*z*, and C_3_D_4_^+^ at 44 *m*/*z*. Additional peaks are also observed at 41 *m*/*z* that corresponds to C_3_HD_2_^+^, which has formed from H-D swapping between C_3_D_4_^+^ and ambient water molecules within the chamber, and at 57 *m*/*z*, 61 *m*/*z* and 62 *m*/*z* corresponding to CaOH^+^ and CaOD^+^ ions. The ^44^CaOH^+^ ions formed through the reaction of ^44^Ca^+^ with H_2_O, and through collisions with C_3_D_4_, eventually formed ^44^CaOD^+^.

To better understand the origin of C_2_O^+^ products produced in reactions *only* with O_2_^+^ (*v* = 2, 3) and not O_2_^+^ (*v* = 0), a portion of the potential energy surfaces (PESs) for the reactions between ground-state O_2_^+^ and the two C_3_H_4_ isomers were calculated using KinBot to search for barrierless pathways to the observed products.^48^

The portion of the PES that shows a possible pathway from reactants to C_2_O^+^ is shown in Figure 3 (propyne) and Supporting Information Figure 2 (allene). The energies of the extrema on the surface were zero-point energy corrected and are displayed relative to the reactants’ energy at infinite separation. It should be noted that unlike higher-pressure systems where collisional stabilization can remove energy from the reaction complex, the single-collision conditions employed here preclude such energy loss. As a result, the total energy of the complex remains equal to its initial energy, preventing it from becoming trapped in local minima and allowing it to readily traverse any submerged barriers on the potential energy surface.

**Figure 3:**
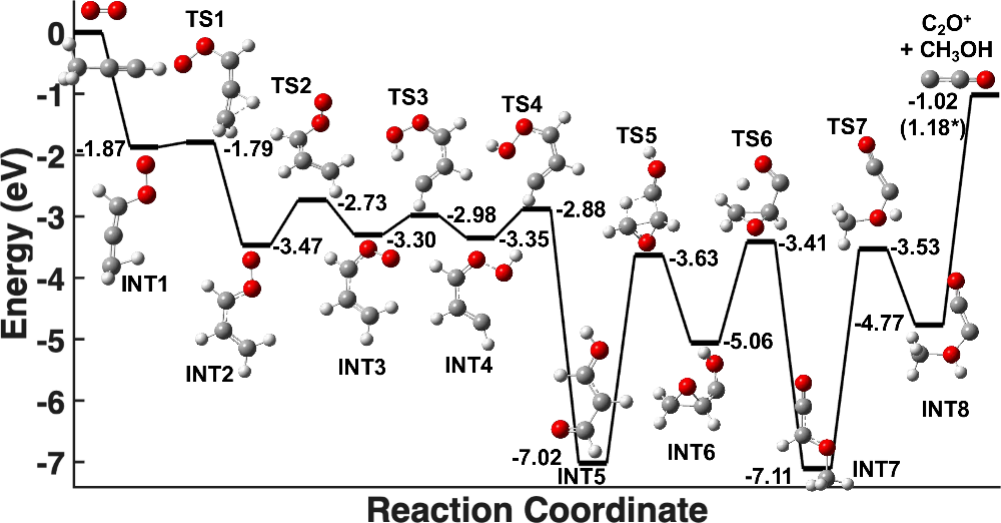
Potential energy surface for the production of C_2_O^+^ in the reaction between O_2_^+^ (*v* = 0) and propyne (H_3_C_3_H). All structures were optimized at the MP2/aug-cc-pVTZ level of theory. Single-point energies of these structures were calculated at the CCSD­(T)-F12/cc-pVDZ-F12 and have been zero-point energy corrected at the MP2/aug-cc-pVTZ level. All energies are shown relative to reactant energy at infinite separation, with INT1 corresponding to the first stationary point following complex formation. Note that two values are reported for the production of C_2_O^+^, one from CCSD­(T)-F12 and the asterisked value computed using values from the ATcT database.^49,50^ See the Computational Methods section for more explanation.

The propyne surface begins with a moderately exothermic (1.87 eV) attachment of O_2_^+^ onto C_3_H_4_ at INT 1. After this initial attachment, the complex can traverse the surface sampling various orientations of the initial attachment complex, including two H atom transfers, before the O–O bond is broken at TS 4. The breaking of the O–O bond and the progression to INT 5 is highly exothermic (∼4 eV exothermic relative to TS 4). After the O–O bond has been severed, the complex can progress through the surface by a series of low-submerged-barrier proton transfers. These proton transfers result in the complex having rearranged into INT 8, where the complex dissociates into C_2_O^+^ and the neutral coproduct CH_3_OH.

The calculated potential energy surfaces revealed that production of C_2_O^+^ (as well as the production of c-C_3_H_3_^+^ and C_3_H_4_^+^, as shown in Supporting Information Figure 3) is exothermic and barrierless for the reactions between O_2_^+^ (*v* = 0) and both C_3_H_4_ isomers. KinBot also discovered over 200 energetically allowed product combinations (with 22 unique mass-to-charge ratios), including all of those observed experimentally. Using just thermodynamic arguments with the results of the KinBot calculations, one would conclude that C_2_O^+^ should be a very minor product as the 127th lowest-energy product, and other products that we did not detect experimentally, such as C_2_H_4_^+^ and C_2_H_3_OH^+^, should have been more prevalent. The observation of C_2_O^+^ and the absence of many products predicted to be more abundant demonstrate that quantum dynamical effects must be at play in these reactions. Such dynamical effects are not uncommon^29,51–54^ and have been observed to prevent barrierless reactions from occurring.^51,52^

Intuitively, one could argue that production of C_2_O^+^ was facilitated in the excited-state reactions because the internal reaction coordinate at TS 4 on the C_2_O^+^ formation PES is directed along the O–O bond. However, for this facilitation to occur, vibrational energy had to remain localized in the O–O bond long enough for this reaction to take place, i.e., until TS 4 is reached. While vibrational energy can, in principle, be removed from a molecule in a variety of ways, including through collision quenching, radiative decay, or intermolecular energy transfer, several of these are unlikely in this particular system. Collisional quenching of excited vibrational modes is not present because of the ultrahigh-vacuum environment of the experiment, which leads to single collision conditions. Radiative decay of vibrational excitation occurs on time scales that are not relevant here; neither O_2_^+^ nor the transient reaction complex has enough time to radiate before the complex dissociates into products. Here, there are two possible modes of energy transfer that could have redistributed the O_2_^+^’s initial vibrational energy, a vibration-vibration energy transfer (VVET) during the initial collision and intramolecular vibration redistribution (IVR) during complex formation.

VVET is known to be most efficient when the two colliding partners have vibrational energy spacings that are similar. For example, collisions between vibrationally excited CO_2_ and N_2_ have been shown to exhibit an order-of-magnitude decrease in energy-transfer efficiency when ^14^N_2_ (with an energy mismatch of approximately 18 cm^–1^) is replaced by ^15^N_2_ (with an energy mismatch of approximately 97 cm^–1^).^55^ For the collisions considered here, the energy of one quanta of the O–O stretch in O_2_^+^ (ν̃ = 1860 cm^–1^, *v* = 2–1) is substantially mismatched with the nearest energetically accessible vibrational modes of allene (H_2_C_3_H_2_) and propyne (H_3_C_3_H), with energy differences of approximately 433 and 424 cm^–1^, respectively.^56–58^ Such large energy mismatches make VVET unlikely. Moreover, even if VVET were to occur, the transfer of a single vibrational quantum would not fully quench the excitation of the O–O stretch; instead, multiple sequential quanta would need to be transferred to prevent vibrational energy from remaining localized in the bond during the initial collision and complex formation.

Although multiquantum VVET has been observed in systems with high vibrational excitation levels (*v* > 15),^59^ single-quantum transfer processes are nearly an order-of-magnitude more probable for low vibrational excitations (*v* < 5).^60^ Consequently, given that the highest excitation involved in the present reactions was *v* = 3, it is reasonable to conclude that, if VVET occurred at all, it was dominated by single-quantum transfer processes. Thus, following the initial collision between O_2_^+^ and C_3_H_4_, one or two, and potentially even three, vibrational quanta likely remained localized in the O–O stretch. A key question then arises: does this vibrational energy remain localized in the O–O bond during complex formation, or was it rapidly redistributed throughout the reaction complex via IVR?

An estimate for the efficiency of IVR in this reaction can be made based on estimations of the reaction complex’s lifetime and comparisons of this lifetime to typical times associated with IVR. The lifetime of the reaction complex can be roughly approximated through the use of statistical models such as Rice–Ramsperger–Kassel–Marcus (RRKM) theory or transition-state theory.^61,62^ While such calculations assume a thermal distribution of states over the course of the reaction as a result of rapid IVR rates, they can be useful for providing an order-of-magnitude estimate for the complex’s lifetime for systems not in thermal equilibrium, as was the case here.

To estimate this lifetime, we performed a simple unimolecular decomposition transition-state theory calculation. In this calculation, the highest energy transition-state structure to C_2_O^+^ production (TS 1, in Figure 3) was chosen so as to maximize the complex’s estimated lifetime, and the reaction complex’s initial structure (INT 1, in Figure 3) was chosen as the predecomposition molecule. These two structures were the only structures used in this calculation. Using these conditions, the reaction complex’s lifetime was estimated to be ∼500 fs. This estimated complex lifetime is an order of magnitude shorter than instances of fast IVR that have been measured to occur over ∼8 ps;^63,64^ however, it has been found that IVR times typically lie in the 20–90 ps range.^65–67^ The difference in these time ranges suggests that IVR could not occur fully before the complex dissociated into the final products. This implies that even in the unlikely case that O_2_^+^ transferred 1 quanta of vibrational energy during the initial collision with C_3_H_4_, the leftover vibrational energy remained localized in the O–O stretch, as it would be unable to couple into the complex’s vibrational modes before dissociation occurred. It is reasonable to conclude that this localization facilitated the breaking of the O–O bond at TS 4 (Figure 3) of the calculated potential energy surface and ultimately led to the formation of C_2_O^+^. However, this qualitative explanation rationalizes only the observation of C_2_O^+^ production. This explanation does not clarify why other products that require breaking the O–O bond, such as C_2_H_3_OH^+^, are not produced. To understand the full extent of how dynamics influences this particular reaction, one would need to perform quantum dynamics simulations on the complete PES. Such an endeavor is beyond the scope of this work, but we hope that our results spur others to explore the dynamics of this complex system.

The results presented here confirm that C_2_O^+^ is a product in the reactions between O_2_^+^ (*v* = 2, 3) and C_3_H_4_ and demonstrate that the production of C_2_O^+^ is governed by dynamical effects rather than just by energetics. While we presented qualitative arguments for how these dynamical effects may appear, a more quantitative argument based on quasi-classical trajectories or other methods is necessary but would be quite challenging to compute based on the number of atoms involved.

Regardless of the complete details of how dynamics directs the reaction complex to one product over another, it is clear from the experimental data that the pathway to C_2_O^+^ is effectively activated by vibrational excitation of O_2_^+^. The result that the preparation of O_2_^+^ in a vibrationally excited state enables formation of an otherwise unobserved product demonstrates the potential for controlling C_2_O^+^ production in this reaction. In principle, the branching ratio for C_2_O^+^ might be further enhanced, possibly to the point of exclusive formation, by preparing O_2_^+^ in higher vibrational states. Exploration of this possibility, as well as the broader role of O_2_^+^ vibrational excitation in other reactions, would be well suited to future studies, particularly given the long lifetimes of the excited vibrational states of O_2_^+^. Similar explorations could be made with other homonuclear diatomics and may reveal interesting chemical behaviors and control.

Although full quantum-state control of the O_2_^+^ + C_3_H_4_ reaction has not yet been realized, this work represents a meaningful advance toward that goal. The results highlight substantial opportunities for probing quantum-state-dependent reactivity and point toward a future in which chemical reaction outcomes can be fully controlled at the quantum level.

## Methods

### Experimental Section

Reactions were conducted using our custom-built ion-trapping apparatus, which has been described previously in depth.^68^ In short, this apparatus consists of a linear, quadrupole ion trap held within an ultrahigh-vacuum environment (∼10^–10^ Torr). Ca^+^ ions were loaded into the trap by nonresonantly photoionizing (∼7 mJ/pulse, 355 nm, 10 Hz) an effusive source of Ca in the center of the ion trap. These trapped Ca^+^ ions were then laser cooled to sub-Kelvin temperatures by using the doubled output of a Ti-Sapph laser (∼397 nm, 2 mW) and a diode laser (∼866 nm, 2 mW). O_2_^+^ ions were then cotrapped with the Ca^+^ ions by resonantly photoionizing a supersonic molecular beam of O_2_ in He (2% mixture) in the center of the trap. Two (2 + 1) REMPI schemes at ∼301 and ∼288 nm were used to form O_2_^+^ ions predominantly in the ground or vibrationally excited states (*v* = 2, 3), respectively.^35–38^

The O_2_^+^ ions were then sympathetically cooled by the laser-cooled Ca^+^ ions to secular temperatures ∼1 K. As the ions cooled, they self-arranged into a pseudocrystalline structure known as a Coulomb crystal. Within the Coulomb crystal, the spacing between ions is around ∼10 μm due to the Coulomb repulsion. Consequently, only the translational motion is sympathetically cooled, while the vibrational and rotational state populations remain unperturbed by the cotrapped ions.

After the O_2_^+^ ions had been trapped and cooled, reactions were initiated by admitting either neutral allene (H_2_C_3_H_2_) or propyne (H_3_C_3_H) into the chamber through a pulsed leak valve at a constant pressure (∼1.2 × 10^–9^ Torr for the ground-state reactions and ∼0.3 × 10^–9^ Torr for the excited-state reactions) for variable periods of time (0–350 s). At various reaction times, the contents of the trap were ejected into a time-of-flight mass spectrometer (TOF-MS) to measure the number of ions at each *m*/*z*. A new Coulomb crystal was loaded for each measurement. Measurements at each time point were repeated >7 times, and the mean and standard error of the mean were plotted and globally fit, with the following chemical equations and pseudo-first-order rate equations (where allene and propyne are treated in excess), as a function of reaction time to produce the observed reaction curves.

4 $${O_{2}}^{+}+C_{3}H_{4}\overset{k_{1}}{\to }C_{3}{H_{3}}^{+}+{\mathrm{HO}}_{2}$$

5 $${O_{2}}^{+}+C_{3}H_{4}\overset{k_{2}}{\to }C_{3}{H_{4}}^{+}+O_{2}\overset{C_{3}H_{4}}{\to }\mathrm{Secondary}\;\mathrm{Products}$$

6 $$\frac{d\left({O_{2}}^{+}\right)}{dt}=-\left(k_{1}+k_{2}\right)\times {O_{2}}^{+}\left(t\right)$$

7 $$\frac{d\left(C_{3}{H_{3}}^{+}\right)}{dt}=k_{1}\times {O_{2}}^{+}\left(t\right)$$

8 $$\frac{d\left(C_{3}{H_{4}}^{+}+\mathrm{Secondary}\mathrm{Products}\right)}{dt}=k_{2}\times {O_{2}}^{+}\left(t\right)$$

where *k*_1_ and *k*_2_ correspond to fitted pseudo-first-order rate constants for production of C_3_H_3_^+^ and C_3_H_4_^+^, respectively, and are reported in Supporting Information Table 1. As C_3_H_4_^+^ shares a mass channel with the most abundant isotope of Ca^+^, direct observation of C_3_H_4_^+^ resulted in large uncertainties from the fitting routine originating from fluctuations in Ca^+^ loading. These fluctuations from Ca^+^ (±50 ions) were avoided by indirectly monitoring the C_3_H_4_^+^ product channel by instead fitting to the directly observed growth of secondary products from the reactions of C_3_H_4_^+^ with additional neutral C_3_H_4_ molecules (with total fluctuations of ∼5 ions). While this approximation does not capture the short reaction time behavior of C_3_H_4_^+^, it captured the C_3_H_4_^+^ branching in the long reaction time limit.

### Computational Methods

Our goal in this work was to identify feasible pathways to the observed products and not to provide a complete and accurate kinetic characterization of the processes at play, especially given the experimental results that suggest that dynamics dominate the branching between the open channels.

To identify feasible pathways, potential energy surfaces (PES) for the reaction between O_2_^+^ (*v* = 0) and C_3_H_4_ isomers were explored using KinBot to search for barrierless (i.e., submerged relative to the reactants) pathways to products. KinBot has been described in depth previously,^48,69^ and only a brief description is provided here. KinBot is an open-source chemical kinetics workflow code that can explore and characterize reactive potential energy surfaces, typically with the aim to predict reaction rate constants. KinBot calculations begin with a geometry optimization of a specified “reactant” molecule, which in this study is the [O_2_–C_3_H_4_]^+^ reaction complex. After this optimization, KinBot generates saddle point guess structures by systematically modifying the initial geometry via a series of constrained optimizations, as dictated by reaction templates. These guess structures are then optimized to true saddle points, and if the optimization is successful and the structure’s energy is below a user-defined threshold, intrinsic reaction coordinate (IRC) calculations are used to ensure the connectivity of the saddle to the reactant and to identify the product(s). If the reaction product is an isomer of the original well, then a new exploration is started by the code until all connected and valid wells are found.

KinBot uses various levels of theory in its workflow. The exploration phase is typically done at a low level of theory, which was L1 = UB3LYP/6-31+G­(d) in this work. Then, the structures are refined at a higher level of theory to obtain better geometries and rovibrational properties, L2. Finally, if desired, the energies can be further refined using single-point energy corrections, L3. For L2 and L3, we used various methods to better understand the underlying uncertainties for the reaction paths of interest. The reported energies (shown in Supporting Information Tables 2 and 3) are at UMP2/aug-cc-pVTZ, ωB97X-D/6-311++G­(d,p), and CCSD­(T)-F12a/cc-pVDZ-F12//UMP2/aug-cc-pVTZ levels of theory. All intermediates and transition states were assumed to be doublets on the C_3_H_4_O_2_^+^ PES. O_2_^+^ is assigned *D*_2*h*_ symmetry in Molpro 2023,^70,71^ which can use only Abelian point groups. The ground state of O_2_^+^ is ^2^Π_*g*_, which translates into B_2g_ or B_3g_ in Molpro. The calculated energies for these two states are degenerate, which is in line with the underlying *D*_2*h*_ symmetry. C_2_O^+^ is also linear but not centrosymmetric (its connectivity is C–C–O); therefore, its Abelian group is *C*_2*v*_. The ground state of C_2_O^+^ is the ^2^Π state,^72^ correlating with the degenerate (for this system) B_1_ and B_2_ symmetries. However, unlike for O_2_^+^, the CCSD­(T)-F12 method yields two nondegenerate states, of which we report the lower one. A more accurate calculation would involve a multireference method instead of the single-reference CCSD­(T)-F12 method, which further requires careful choices of other species for proper referencing of relative energies. To reduce some of the uncertainties introduced by the single-reference method for C_2_O^+^, we report both a CCSD­(T)-F12 energy and a value determined using the ATcT v.1.220 database for comparison.^49,50^ For the DFT and MP2 calculations we used Gaussian 16,^73^ while for the CCSD­(T)-F12 energies we used Molpro.

Using the above workflow, we were able to determine an expansive potential energy surface for this reaction. We found a very large number of pathways, with >150 wells and >200 bimolecular product channels at L1, all satisfying the energetic criterion, which was that no barrier shall be above the energy of the starting species. The reason for the vastness of the PES is that the reactants initially form very strong bonds at the addition step. After this attachment, the PES descends rapidly by breaking the O–O bond, resulting in several wells on the PES that are 6 eV or deeper relative to the reactants. We analyzed the large reaction network using our postprocessing tool, PESViewer,^74^ which allowed us to find feasible pathways between any two species on the surface and explore some pathways manually.

## Supplementary Material



## Notes

Table of fitted rate constants, tables of calculated energetics, normalized ion signal, and potential energy surfaces (PDF)
