# Supplementary material for: Vibrational Quantum-State-Controlled Reactivity in the O2 + + C3H4 Reaction
Source: J Phys Chem Lett. 2026 Apr 6;17(15):4621–9. doi: 10.1021/acs.jpclett.6c00425 (PMC13093674; doi:10.1021/acs.jpclett.6c00425)
Supplement: Supplementary file 1 [file jz6c00425_si_001.pdf]

Supporting Information for Publication

Vibrational Quantum-State-Controlled Reactivity in the $O_2{}^+ + C_3H_4$ Reaction

C. Zagorec-Marks,[*,†,‡] G. S. Kocheril,[†,‡] T. Kieft,[†,‡] O. A. Krohn,[¶,†,‡] C. Martí,[¶] T. P. Softley,[§] J. Zádor,[¶] and H. J. Lewandowski[*,†,‡]

†*Department of Physics, University of Colorado, Boulder, CO 80309, USA*

‡*JILA, National Institute of Standards and Technology and the University of Colorado, Boulder, CO 80309, USA.*

¶*Combustion Research Facility, Sandia National Laboratories, Livermore, CA 94550, USA*

§*School of Chemistry, University of Birmingham, Edgbaston, B15 2TT, UK*

E-mail: chase.zagorec-marks@colorado.edu; lewandoh@colorado.edu

Table S1: Fitted rate constants for the $O_2^+(v=i) + C_3H_4$ reactions. The uncertainties represent statistical measurement uncertainties from the fits only and do not account for any systematic uncertainties in the measurement of neutral concentration that arise because Bayard-Alpert hot cathode ion gauges have reduced accuracy below 10^{-8} Torr.[1] Note that reported rate constants for the production of $C_3H_4^+$ in the ground-state reactions are approximations from fitting to secondary product growth, and thus carry additional uncertainty in addition to underestimating the rate.

Reaction	k ($10^{-10} \frac{cm^3}{s}$)
$O_2^+(v=0) + H_3C_3H \rightarrow c - C_3H_3^+ + HO_2$	3.3 ± 0.6
$O_2^+(v=0) + H_3C_3H \rightarrow C_3H_4^+ + O_2$	2.6 ± 0.5
$O_2^+(v=0) + H_2C_3H_2 \rightarrow c - C_3H_3^+ + HO_2$	5.0 ± 0.9
$O_2^+(v=0) + H_2C_3H_2 \rightarrow C_3H_4^+ + O_2$	0.9 ± 0.3
$O_2^+(v=2,3) + H_3C_3H \rightarrow c - C_3H_3^+ + HO_2$	7.4 ± 0.8
$O_2^+(v=2,3) + H_3C_3H \rightarrow C_3H_4^+/C_2O^+ + O_2/CH_3OH$	4 ± 1
$O_2^+(v=2,3) + H_2C_3H_2 \rightarrow c - C_3H_3^+ + HO_2$	7.0 ± 0.7
$O_2^+(v=2,3) + H_2C_3H_2 \rightarrow C_3H_4^+/C_2O^+ + O_2/CH_3OH$	1.8 ± 1.0

References

(1) Jousten, K. Gauges for fine and high vacuum. *CAS- CERN Accelerator School: Vaccum in Accelerators* **2007**, 65–86.

(2) Ruscic, B.; Pinzon, R. E.; Morton, M. L.; von Laszevski, G.; Bittner, S. J.; Nijsure, S. G.; Amin, K. A.; Minkoff, M.; Wagner, A. F. Introduction to Active Thermochemical Tables: Several "Key" Enthalpies of Formation Revisited. *J. Phys. Chem. A* **2004**, *108*, 9979–9997.

(3) Ruscic, B.; Pinzon, R. E.; von Laszewski, G.; Kodeboyina, D.; Burcat, A.; Leahy, D.; Montoy, D.; Wagner, A. F. Active Thermochemical Tables: Thermochemistry for the 21st Century. *J. Phys.: Conf. Ser.* **2005**, *16*, 561–570.

Table S2: Calculated energetics for the formation of C_2O^+ in the reactions of ${O_2}^+$ with propyne (H_3C_3H) as shown in Main Text Fig. 4. All structures were optimized at the UMP2/aug-cc-pVTZ level of theory. Energetics calculations were run at three separate levels of theory: UωB97X-D/6-311++G(d,p), UMP2/aug-cc-pVTZ, and CCSD(T)-F12a/cc-pVDZ-F12//UMP2/aug-cc-pVTZ. DFT calculations have been zero-point energy corrected with the same method. MP2 and CCSD(T)-F12 calculations have been zero-point energy corrected with the MP2 method. All energies are displayed relative to the initial reactants at infinite separation.

PES Extrema	DFT (eV)	MP2 (eV)	CCSD(T)-F12 (eV)
Propyne INT1	-2.46	-1.04	-1.87
Propyne TS1	-2.38	-0.96	-1.79
Propyne INT2	-3.69	-2.65	-3.47
Propyne TS2	-3.36	-1.84	-2.73
Propyne INT3	-3.68	-2.47	-3.30
Propyne TS3	-3.33	-2.12	-2.98
Propyne INT4	-3.82	-2.57	-3.35
Propyne TS4	-3.29	-2.09	-2.88
Propyne INT5	-7.60	-6.17	-7.02
Propyne TS5	-4.36	-3.39	-3.63
Propyne INT6	-5.52	-4.69	-5.06
Propyne TS6	-3.82	-3.06	-3.41
Propyne INT7	-7.64	-6.76	-7.11
Propyne TS7	-4.03	-3.15	-3.53
Propyne INT8	-5.25	-4.28	-4.77
C_2O^+ + CH_3OH	-1.43	-0.57	-1.02

Table S3: Calculated energetics for the formation of C_2O^+ in the reactions of ${O_2}^+$ with allene ($H_2C_3H_2$) as shown in Supporting Information Fig. S2. All structures were optimized at the UMP2/aug-cc-pVTZ level of theory. Energetics calculations were run at three separate levels of theory: UωB97X-D/6-311++G(d,p), UMP2/aug-cc-pVTZ, and CCSD(T)-F12a/cc-pVDZ-F12//UMP2/aug-cc-pVTZ. DFT calculations have been zero-point energy corrected with the same method. MP2 and CCSD(T)-F12 calculations have been zero-point energy corrected with the MP2 method. All energies are displayed relative to the initial reactants at infinite separation.

PES Extrema	DFT (eV)	MP2 (eV)	CCSD(T)-F12 (eV)
Allene INT1	-2.82	-1.58	-2.32
Allene TS1	-2.50	-1.37	-2.04
Allene INT2	-3.65	-2.83	-3.51
Allene TS2	-3.32	-2.02	-2.77
Allene INT3	-3.64	-2.66	-3.34
Allene TS3	-3.29	-2.30	-3.02
Allene INT4	-3.78	-2.76	-3.39
Allene TS4	-3.25	-2.27	-2.92
Allene INT5	-7.56	-6.36	-7.05
Allene TS5	-4.32	-3.57	-3.67
Allene INT6	-5.48	-4.88	-5.09
Allene TS6	-3.78	-3.25	-3.45
Allene INT7	-7.60	-6.94	-7.15
Allene TS7	-3.99	-3.33	-3.57
Allene INT8	-5.21	-4.47	-4.81
C_2O^+ + CH_3OH	-1.39	-0.75	-1.06

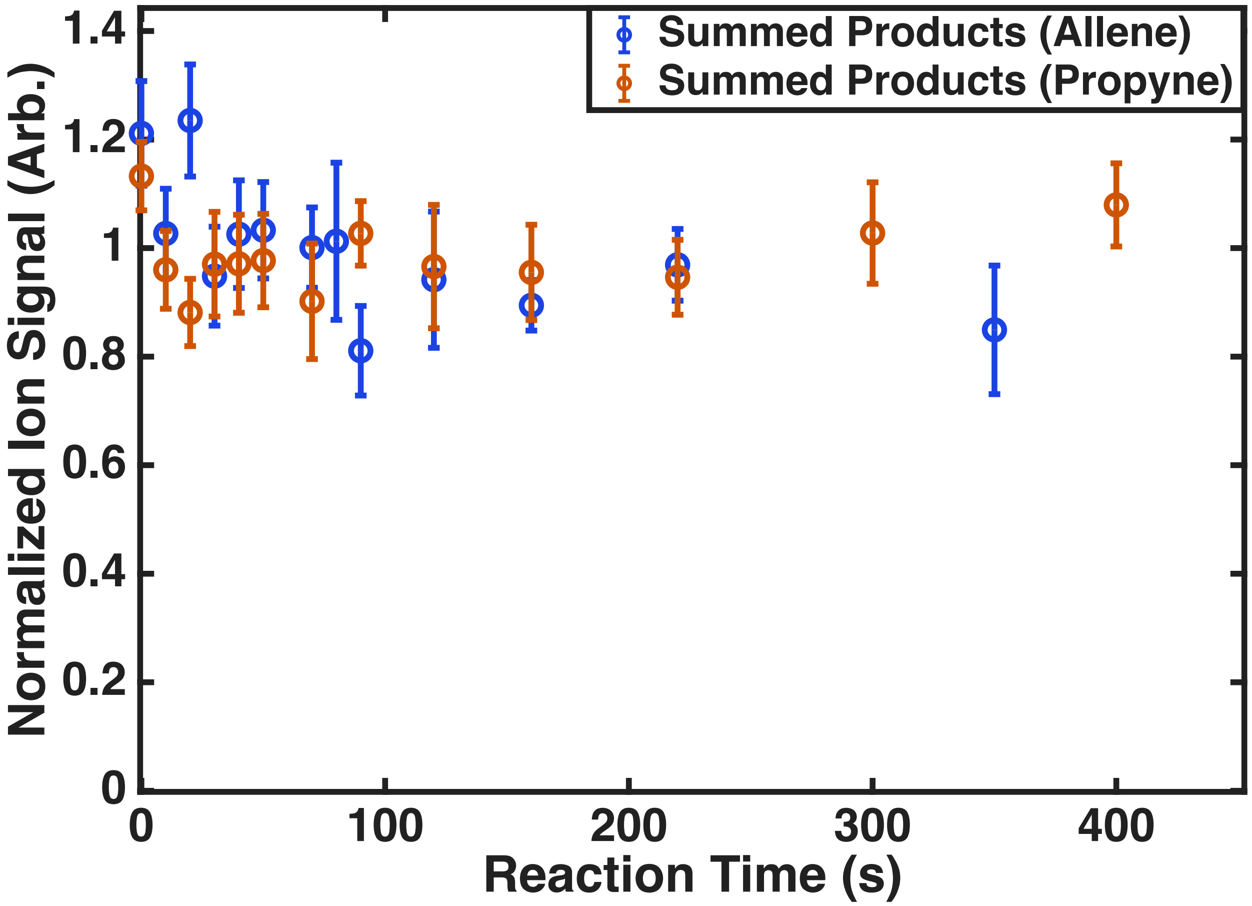

Figure S1: Normalized ion signal obtained from the sum of detected O_2^+, $C_3H_3^+$, $C_6H_5^+$, and $C_6H_7^+$ ions throughout the reaction of O_2^+ with allene (blue) and propyne (orange). Sums have been normalized to the fitted initial values of O_2^+ for their respective reaction series.

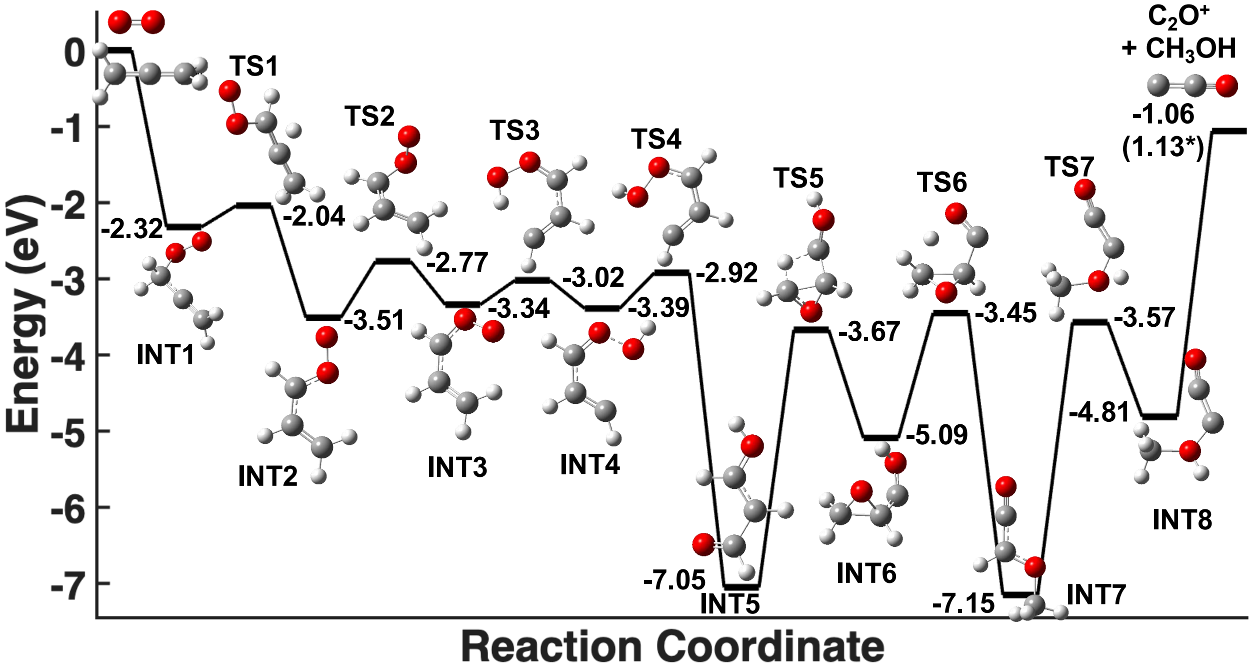

Figure S2: Potential energy surface for the production of C_2O^+ in the reaction between ${O_2}^+(v = 0)$ and allene ($H_2C_3H_2$). All structures were optimized at the UMP2/aug-cc-pVTZ level of theory. Single-point energies of these structures were calculated at the CCSD(T)-F12/cc-pVDZ-F12 and have been zero-point energy corrected at the UMP2/aug-cc-pVTZ level. All energies are shown relative to reactants energy at infinite separation, with INT1 corresponding to the first stationary point following complex formation. Note that two values are reported for the production of C_2O^+, one from CCSD(T)-F12 and the asterisked value computed using values from the ATcT database.[2,3] See Computational Methods for more explanation.

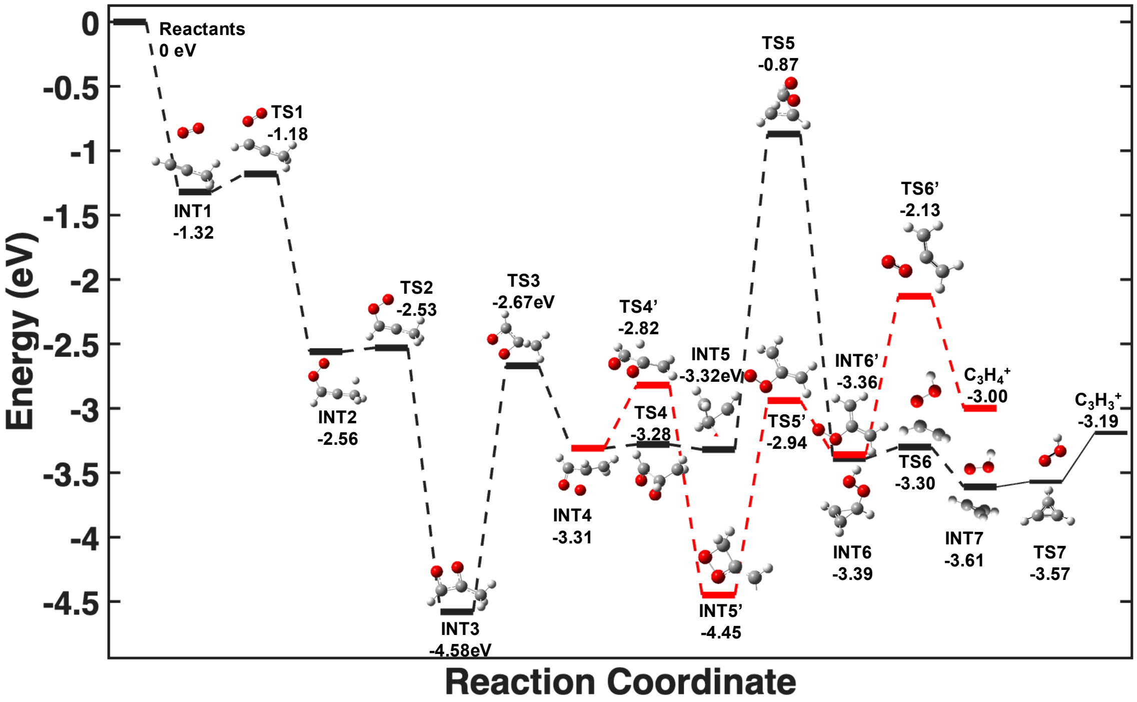

Figure S3: Potential energy surface for the production of c-$C_3H_3^+$ and $C_3H_4^+$ in the reaction between $O_2^+(v = 0)$ and propyne (H_3C_3H) calculated at the UωB97X-D/aug-cc-pvtz level of theory. All energies have been zero-point energy corrected and are shown relative to reactants energy at infinite separation, with INT1 corresponding to the first stationary point following complex formation. Note that TS 7 is labeled as a transition state; however, it corresponds to a loosely-bound transition state, which is more similar to a saddle point.
